# Correction: Stimulated Bacterial Growth under Elevated *p*CO_2_: Results from an Off-Shore Mesocosm Study

**DOI:** 10.1371/journal.pone.0103694

**Published:** 2014-07-21

**Authors:** The PLOS ONE Staff

## Notice of Republication

This article was republished on July 2nd, 2014, due to an error in the title. The publisher apologizes for this error. Please download this article again to view the corrected title and citation. The originally published, uncorrected article and the republished, corrected article are provided here for reference.

## Supporting Information

File S1 Originally published, uncorrected article.
(PDF)

File S2 Republished, corrected article.
(PDF)

## References

[1] Endres S, Galgani L, Riebesell U, Schulz K-G, Engel A (2014) Stimulated Bacterial Growth under Elevated *p* CO_2_: Results from an Off-Shore Mesocosm Study. PLoS ONE 9(6): e99228 doi:10.1371/journal.pone.0099228 24941307 10.1371/journal.pone.0099228 PMC4062391

