# Supplementary figures and images for: Stimulated Bacterial Growth under Elevated pCO2: Results from an Off-Shore Mesocosm Study
Source: PLoS One. 2014 Jun 18;9(6):e99228. doi: 10.1371/journal.pone.0099228 (PMC4062391; doi:10.1371/journal.pone.0099228)

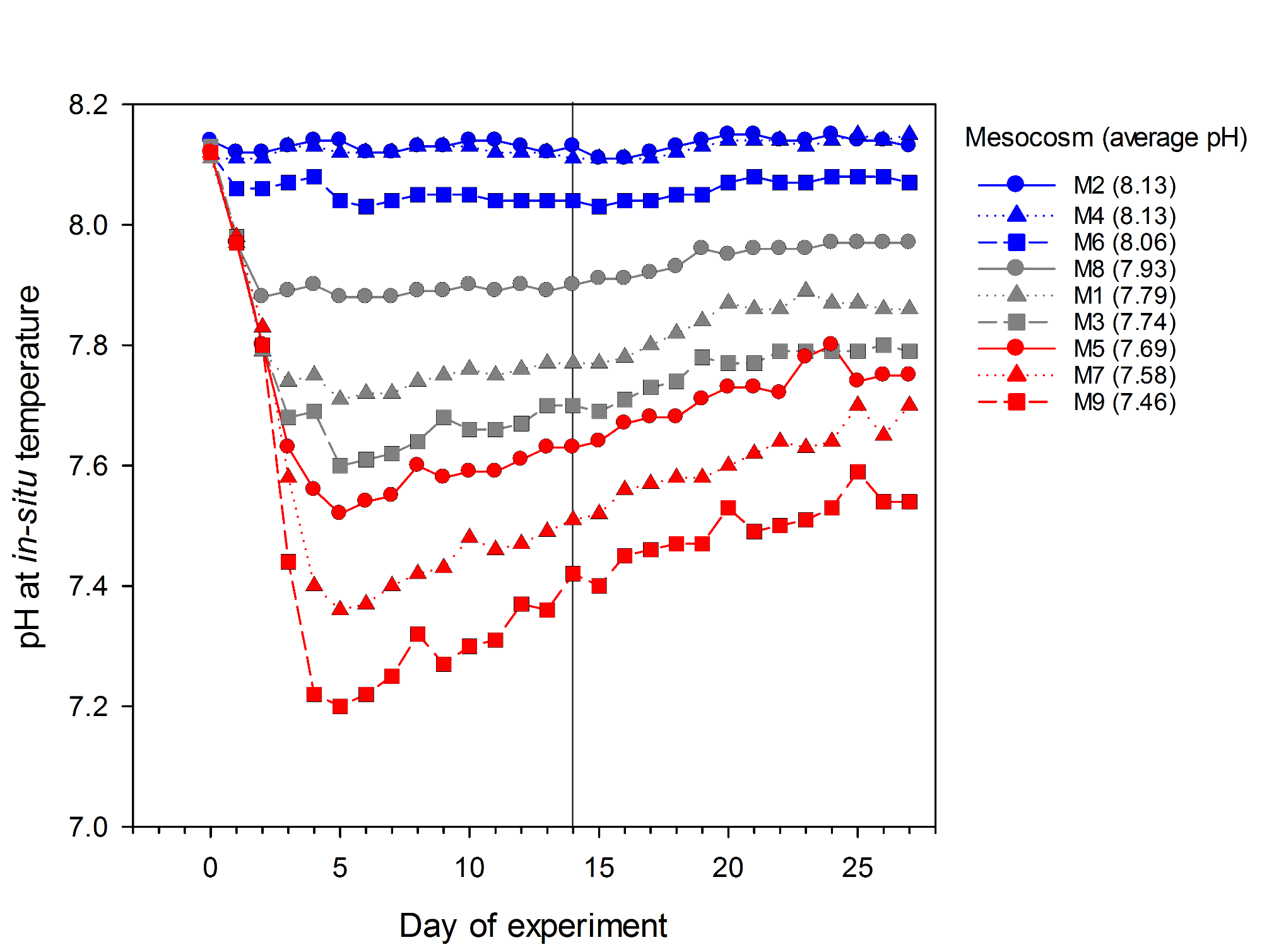

Supplement: Figure S1 — pH values in the nine mesocosms over time. Mesocosms were adjusted until day 5 to target pCO2 levels by stepwise additions of CO2 saturated seawater. Nutrients were added to all mesocosms on day 14. (TIF) [file pone.0099228.s001.tif]

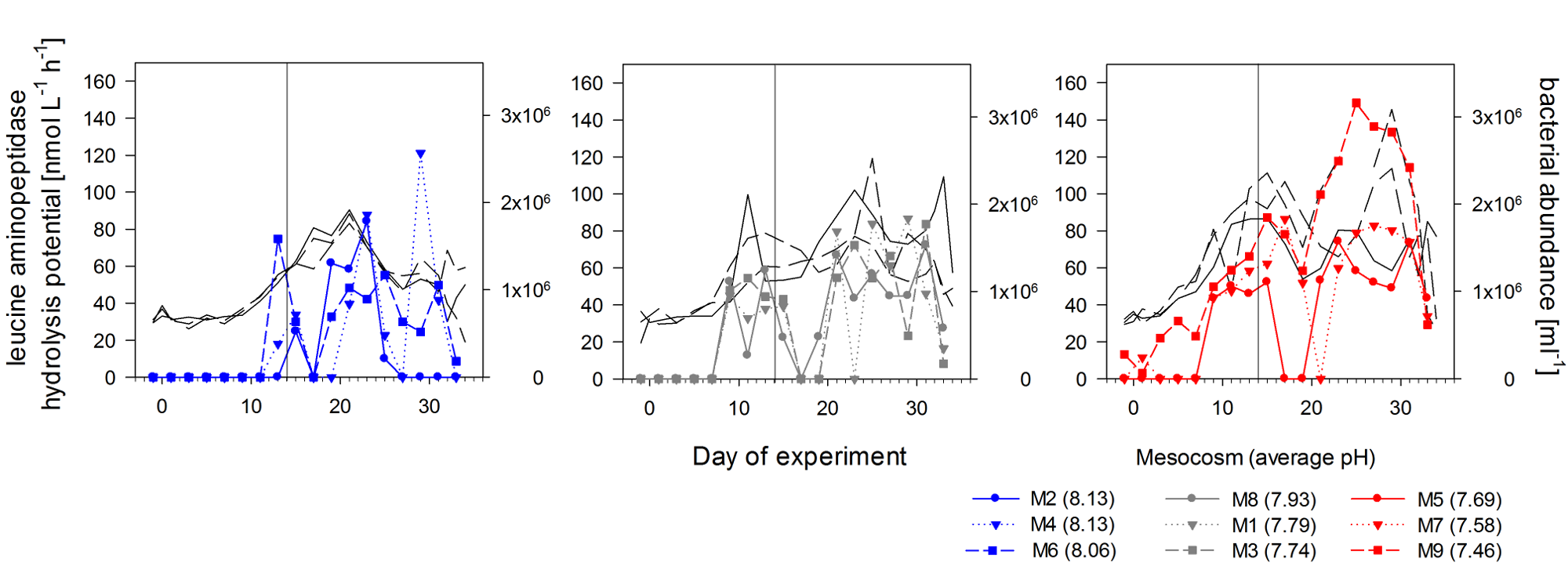

Supplement: Figure S2 — Leucine aminopeptidase (LAP) hydrolysis potential. Temporal development of total LAP hydrolysis potential in the high (blue), intermediate (grey) and low (red) pH mesocosms during the course of the experiment. Black lines indicate bacterial abundances in the corresponding mesocosms. Numbers in brackets give the mean pH value of each treatment over time. Vertical black line indicates the day of nutrient addition. (TIF) [file pone.0099228.s002.tif]

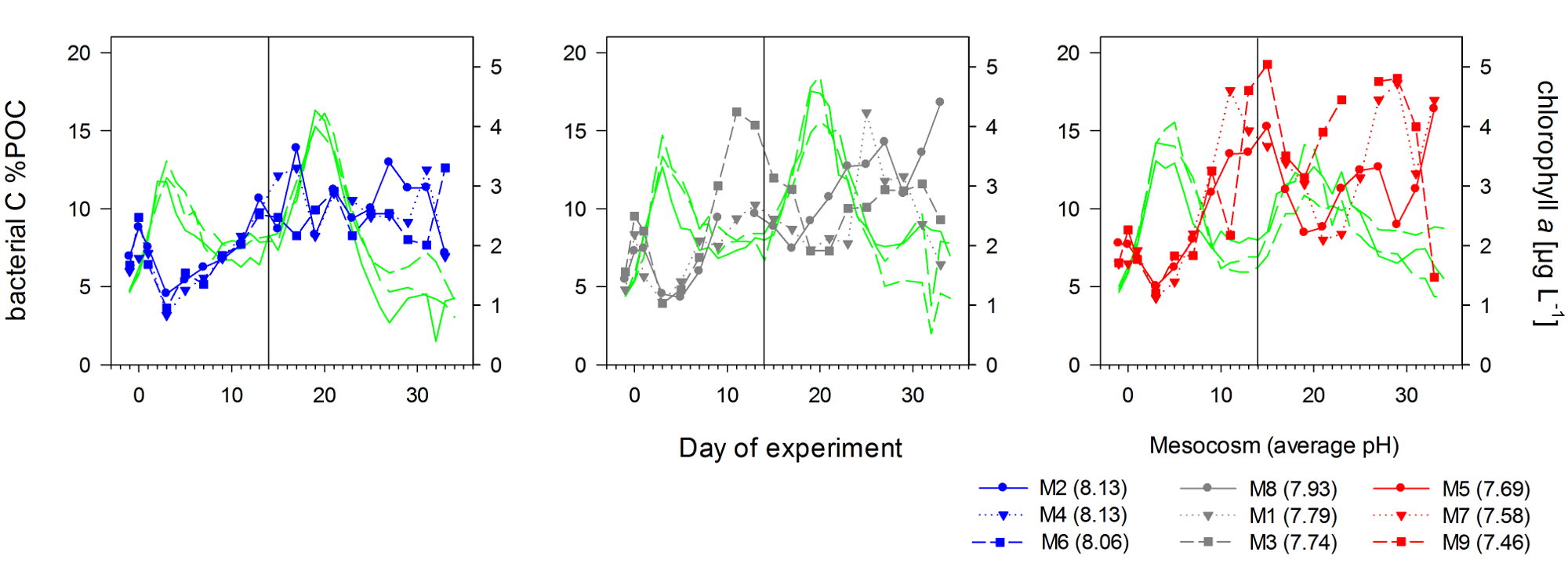

Supplement: Figure S3 — Percentage of bacterial biomass in particulate organic carbon over time in high (blue), intermediate (grey) and low (red) pH mesocosms. Green lines indicate chlorophyll a concentrations in the corresponding mesocosms. Vertical black line indicates the day of nutrient addition. (TIF) [file pone.0099228.s003.tif]
