# Supplementary material for: Correction: Stimulated Bacterial Growth under Elevated pCO2: Results from an Off-Shore Mesocosm Study
Source: PLoS One. 2014 Jul 21;9(7):e103694. doi: 10.1371/journal.pone.0103694 (PMC4105530; doi:10.1371/journal.pone.0103694)
Supplement: File S1 — Originally published, uncorrected article. (PDF) [file pone.0103694.s001.pdf]

OPEN ACCESS Freely available online

PLOS ONE

Stimulated Bacterial Growth under Elevated pCo_2: Results from an Off-Shore Mesocosm Study

Sonja Endres[1,2]*, Luisa Galgani[1,2], Ulf Riebesell[1], Kai-Georg Schulz[1,3], Anja Engel[1]

1 Biological Oceanography, GEOMAR Helmholtz Centre for Ocean Research Kiel, Kiel, Germany, 2 Polar Biological Oceanography, Alfred Wegener Institute Helmholtz Centre for Polar and Marine Research (AWI), Bremerhaven, Germany, 3 Centre for Coastal Biogeochemistry, School of Environmental Science and Management, Southern Cross University, Lismore, Australia

Abstract

Marine bacteria are the main consumers of freshly produced organic matter. Many enzymatic processes involved in the bacterial digestion of organic compounds were shown to be pH sensitive in previous studies. Due to the continuous rise in atmospheric CO_2 concentration, seawater pH is presently decreasing at a rate unprecedented during the last 300 million years but the consequences for microbial physiology, organic matter cycling and marine biogeochemistry are still unresolved. We studied the effects of elevated seawater pCO_2 on a natural plankton community during a large-scale mesocosm study in a Norwegian fjord. Nine Kiel Off-Shore Mesocosms for Future Ocean Simulations (KOSMOS) were adjusted to different pCO_2 levels ranging initially from ca. 280 to 3000 µatm and sampled every second day for 34 days. The first phytoplankton bloom developed around day 5. On day 14, inorganic nutrients were added to the enclosed, nutrient-poor waters to stimulate a second phytoplankton bloom, which occurred around day 20. Our results indicate that marine bacteria benefit directly and indirectly from decreasing seawater pH. During the first phytoplankton bloom, 5–10% more transparent exopolymer particles were formed in the high pCO_2 mesocosms. Simultaneously, the efficiency of the protein-degrading enzyme leucine aminopeptidase increased with decreasing pH resulting in up to three times higher values in the highest pCO_2/lowest pH mesocosm compared to the controls. In general, total and cell-specific aminopeptidase activities were elevated under low pH conditions. The combination of enhanced enzymatic hydrolysis of organic matter and increased availability of gel particles as substrate supported up to 28% higher bacterial abundance in the high pCO_2 treatments. We conclude that ocean acidification has the potential to stimulate the bacterial community and facilitate the microbial recycling of freshly produced organic matter, thus strengthening the role of the microbial loop in the surface ocean.

Citation: Endres S, Galgani L, Riebesell U, Schulz K-G, Engel A (2014) Stimulated Bacterial Growth under Elevated pCo_2: Results from an Off-Shore Mesocosm Study. PLoS ONE 9(6): e99228. doi:10.1371/journal.pone.0099228

Editor: Sam Dupont, University of Gothenburg, Sweden

Received February 5, 2014; **Accepted** May 12, 2014; **Published** June 18, 2014

Copyright: © 2014 Endres et al. This is an open-access article distributed under the terms of the Creative Commons Attribution License, which permits unrestricted use, distribution, and reproduction in any medium, provided the original author and source are credited.

Funding: This work was funded by the BMBF projects BIOACID (Biological impacts of ocean acidification, 03F0608B) and SOPRAN II (Surface Ocean Processes in the Anthropocene, 03F0611C-TP01). This is contribution to the international SOLAS programme. The funders had no role in study design, data collection and analysis, decision to publish, or preparation of the manuscript.

Competing Interests: The authors have declared that no competing interests exist.

* E-mail: sendres@geomar.de

Introduction

Marine bacteria play a central role in the marine carbon cycle [1,2] as they influence the cycling and export of organic matter to the deep sea [1,3]. In the past 200 years, the oceans have absorbed approximately one half of anthropogenic CO_2 emissions [4] leading to a decrease in seawater pH [5]. Little is known on how bacterial communities in the surface ocean might respond to future changes in pCO_2 and pH conditions [6]. Currently, 50 to 96% of net marine primary production is routed into the microbial loop and respired to CO_2 by bacterioplankton [7]. Just a minor fraction of the organic matter produced by photosynthesis in the ocean escapes bacterial respiration and is sequestrated in the deep sea for 100 years and more [8,9]. The efficiency and strength of the biological carbon pump depends upon the balance of organic matter production at the surface (<100 m) and bacterial remineralization and particle dissolution in the surface and mesopelagic (0–1000 m) ocean [10,11]. Small changes in the balance between autotrophic production and heterotrophic degradation processes in the surface ocean, caused e.g. by microbial responses to ocean acidification and warming [12–17], may considerably feedback on atmospheric CO_2 concentrations.

Dissolved polymers, like polysaccharides, can form a gel-like matrix such as transparent exopolymer particles (TEP), which comprise a substantial and highly dynamic fraction of the particulate organic matter pool [18,19]. Furthermore, TEP promote the downward transport of organic matter in the water column and may serve as a food source for bacteria but also act as a substrate for cells to attach and grow [18,20]. So far, it has been found that doubling of the present atmospheric CO_2 concentration slightly stimulates the rate of photosynthesis in most marine algae tested [21–23] while the rate of extracellular organic carbon production and formation of TEP significantly increases [12,16] potentially affecting organic matter pools in the future ocean. Bacterial growth is regulated by abiotic factors (e.g. temperature) but also largely by the availability and accessibility of freshly produced organic matter [24]. Consequently, changes in the TEP pool may influence bacterial hydrolysis and growth; on the other hand, changes in bacterial hydrolysis rates may influence the biogeochemical fate of organic matter [25].

Marine bacteria are very efficient in carbon and nutrient acquisition and compete with phytoplankton for inorganic and organic substrates [26,27]. Small, labile molecules (<1000 Da) such as mono- and disaccharides and amino acids are easily assimilated into bacterial cells making them good indicators of phytoplankton release and microbial degradation processes. In order to utilize the high-molecular weight (>1000 Da) dissolved organic matter (DOM) fraction, bacteria produce extracellular enzymes that degrade polymers into smaller compounds [28]. This DOM fraction is turned over faster, compared to smaller compounds, as it contains carbon- and energy-rich substrates such as proteins and polysaccharides [29,30]. Microbial processing may also modify the molecular structure of DOM forming organic compounds whose chemical structures are largely unknown and may resist further degradation [31]. The production and modification of this refractory DOM via heterotrophic microbial processes is another potential sink for CO_2 in the ocean [32] and has been termed the 'microbial carbon pump' [33].

As hydrolytic extracellular enzymes play a central role in organic matter remineralisation [34], abiotic and biotic factors controlling enzyme activities need to be understood. Generally, enzyme activities show strong pH dependency because changes in hydrogen ion concentration can modify the three-dimensional structure of the active site of an enzyme [35]. It has been shown that protein hydrolysis by the enzyme leucine aminopeptidase may decrease if the sample gets acidified below pH 7.2 [36]. Several laboratory and mesocosm studies determined increased enzymatic hydrolysis rates of proteins [37], polysaccharides [38], organic phosphorous compounds [39], and lipids [40] in natural plankton communities exposed to decreased seawater pH as expected for the near future, suggesting faster element cycling within the microbial loop in the future ocean. Still, the answer whether or not marine bacteria may benefit from acidification by increasing abundance and enzymatic degradation rates is pending [6].

The main goal of our study was to investigate how the combination of direct pH effects (on enzyme activities) and indirect pCO_2 effects (formation of TEP) may affect or even amplify bacterial growth. Therefore, we examined the dynamics of bacterial abundance, TEP, and the activity of one key enzyme in bacterial degradation (extracellular peptidase) in a natural plankton community subjected to various CO_2 concentrations. The large-scale mesocosm set-up used here allows manipulation of environmental factors (here CO_2 and nutrients) and the response of natural plankton communities and several trophic levels under close to natural conditions to be followed continuously.

Methods

Experimental set-up and bloom development

The experiment was conducted using the Kiel Off-Shore Mesocosms for Future Ocean Simulations (KOSMOS) that allow plankton dynamics to be followed over several weeks with minimal disturbance of the water body and under *in-situ* conditions. Experimental perturbations included CO_2 enrichment and nutrient additions. A detailed description of the experimental setup, its deployment, technical features and the sampling methods are described in Riebesell et al. (2013, [41]) and Schulz et al. (2013, [42]). Briefly, nine 25 m-long, free-floating mesocosms with flexible thermoplastic polyurethane bags were deployed, in clusters of three, in the Raunefjord at 60.31°N 5.16°E near Bergen in Southern Norway (Fig. 1) on 30th of April 2011 (day −8). Water depth at the deployment site was between 55 and 65 m. The permit for the operation of the mesocosm facility at the study site was issued by the Port of Bergen. This field study did not involve endangered or protected species.

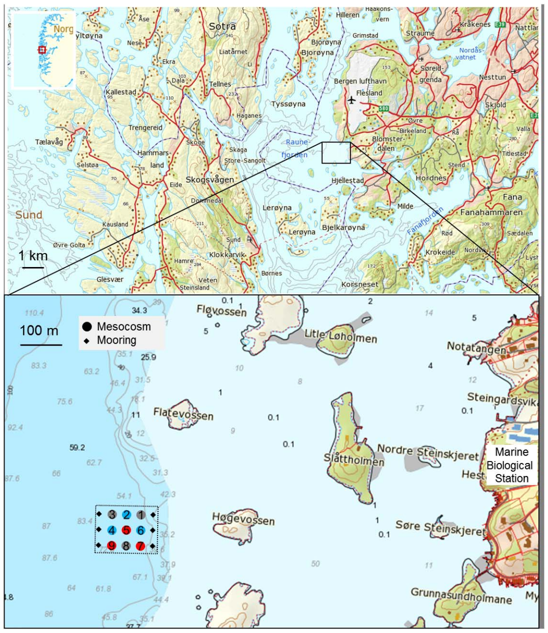

Figure 1. Map of the Raunefjord on the west coast of Norway. Insert shows the study area with the location and orientation of the mesocosm array. Colored circles indicate the position of high (blue), intermediate (grey) and low (red) pH mesocosms. Source of map: The Norwegian Mapping Authority (Kartverket), http://geo.ngu.no/kart/arealisNGU/.
doi:10.1371/journal.pone.0099228.g001

Seven mesocosms were adjusted over five days to target pCO_2 levels of ~400 (M6), 600 (M8), 900 (M1), 1200 (M3), 1300 (M5), 2000 (M7) and 3000 (M9) µatm by stepwise additions of CO_2 saturated seawater. Two mesocosms (M2, M4) were used as control treatments at *in-situ* pCO_2 of approximately 300 µatm. CO_2 treatments were arranged in an assorted design of the mesocosms in relation to each other and the shore to avoid that external variation (e.g. light or salinity gradients) could bias our treatment effects (Fig. 1). After termination of the experiment, one small hole was detected in the bag of M2. We decided to show M2 data in this manuscript as nutrients, chlorophyll (Chl) *a*, TEP concentrations, pH, bacterial abundances, and leucine aminopeptidase activities indicate that the exchange of water during the experiment did not significantly affect these parameters. However, we excluded M2 from statistical analysis.

After CO_2 addition, samples of the entire enclosed water column were taken with a depth integrating water sampler (0–23 m depth, Hydrobios Kiel, Germany) daily for 35 days from all nine mesocosms and the fjord. Sampling started on 7th of May, one day before the first CO_2 addition (termed "day −1"). On day 14, 5 µmol L^{-1} nitrate and 0.16 µmol L^{-1} phosphate were added to the mesocosms. Mesocosms walls were cleaned regularly with a ring-shaped, double-bladed wiper to prevent significant biofilm growth [41].

pH measurements

pH was measured spectrophotometrically with a VARIAN Cary 100 in 10 cm cuvette at 25°C as described in Dickson (2010, [43]) and then recalculated to *in-situ* temperature. pH values are presented on the total scale. The precision was typically better than 0.001 units at high and 0.002 units at low pH.

Chlorophyll a analysis

For chlorophyll *a* (Chl *a*) analysis 250–500 mL samples were filtered onto Whatman GF/F filters. Filters were stored at −80°C for at least 24 h and then homogenized with 90% acetone and glass beads (2 and 4 mm) in a cell mill. After centrifugation, chlorophyll *a* concentrations were determined with a TURNER 10-AU fluorometer as described in Welschmeyer (1994, [44]).

Bacterial abundance and biomass

For bacterial cell counts, 4.5 mL samples were preserved with 200 µL glutaraldehyde (1% v/v final concentration) and stored at −20°C for up to three months until measurement. A stock solution of SybrGreen I (Invitrogen) was prepared by mixing 5 µL of the dye with 245 µL dimethyl sulfoxide (DMSO, Sigma Aldrich). 5 µL of the dye stock solution and 10 µL fluoresbrite microspheres (diameter 0.94 µm, Polysciences) were added to 200 µL of the thawed sample and incubated for 30 min in the dark. The samples were then analysed at low flow rate using a flow cytometer (FACS Calibur, Becton Dickinson) [45]. TruCount beads (Becton Dickinson) were used for calibration and in combination with Fluoresbrite YG microsphere beads (1.00 µm, Polysciences) for absolute volume calculation. Calculations were done using the software program "Cell Quest Pro". Bacterial abundance in the highest pCO_2 mesocosm (M9) on day 25 was exceptionally high (4.5×10^6 cells mL^{-1}) compared to other mesocosms and sampling days. To avoid misinterpretation of a potential CO_2 effect, this data point was excluded from analysis.

Flow cytometry is a fast and highly reproducible method to determine bacterial cell counts. It may detect small bacteria (< 0.2 µm) that are difficult to distinguish with an epifluorescence microscope. Bacteria attached to small, transparent gel particles may also be included. However, it cannot detect large aggregates (>30 µm). Therefore, bacterial cell counts refer mainly to free-living bacterial cells while particle-attached bacteria may be underestimated. Several factors are known to influence the precision of bacteria cell counting. Depending on fixative, storage time and temperature, cells may rapidly decay over time [46,47]. To reduce bacterial cell loss in our study, samples were preserved with glutaraldehyde, which has been shown to be a suitable fixative [48] and superior over formaldehyde regarding the signal intensity of SybrGreen I staining [49]. Furthermore, samples were stored as suggested at −20°C for maximum three months until measurement [46,47].

Bacterial biomass was calculated by multiplying bacterial cell counts with an elemental content of 9 fg carbon $cell^{-1}$ according to Fagerbakke *et al.* [50] who determined the elemental composition of bacteria from the Raunefjord in June and October 1996. Bacterial C content was then compared to particulate organic carbon concentrations (data provided by K. Schulz). The values determined by Fagerbakke *et al.* are relatively low compared to other estimates of bacterial element content and therefore bacterial biomass contribution to particulate organic matter in this study might be underestimated.

Protein hydrolysis potential

The leucine aminopeptidase (LAP) activity is frequently used as an indicator for microbial metabolic processes involved in the mineralization of peptides and proteins [51]. The potential *in situ* activity of the LAP (referred to as 'protein hydrolysis potential') was determined by using the fluorogenic model substrates L-leucine-4-methyl-7-coumarinylamide (MCA) [52]. L-leucine-MCA was added to 180 µl samples and incubated in duplicates for 5–20 h in the dark at 11°C. Six different substrate concentrations ranging from 0 to 150 µM (0, 1, 10, 20, 50 and 150 µM) were tested. Sample fluorescence was measured in microtiter plates with a fluorometer (FLUOstar OMEGA, BMG Labtech, excitation 355 nm, emission 460 nm). Calibration was carried out with a dilution series of MCA. The fluorescent signal of MCA was tested and not affected by pH changes in the range of pH 7.2–8.4. Detection limit for the LAP activity was 2 nmol L^{-1}. Distilled water or sterilized seawater was incubated with substrate at each of the concentrations as a control for background fluorescence and abiotic substrate hydrolysis. The activity of bacterial extracellular enzymes was calculated as the maximum hydrolysis rate (V_{max}; i.e. the maximum rate achieved by the system at saturating substrate concentrations) using the software SigmaPlot 12.0 (Systat). Maximal rates were normalized to bacterial abundances to get cell-specific LAP activities. The Michaelis constant (K_m) is the substrate concentration at which the hydrolysis rate is ½ V_{max} [53]. A low K_m value indicates high enzyme affinity to the substrate while a high K_m value points to lower substrate affinity which means more unspecific binding of molecules to the active site of the enzyme. The V_{max}:K_m ratio is used as an estimate of enzyme efficiency.

Transparent exopolymer particles (TEP)

For photometric analysis of acidic polysaccharide-containing transparent exopolymer particles (TEP), 20 to 60 mL samples were filtered onto 0.4 µm polycarbonate filters, stained with a calibrated Alcian Blue solution and rinsed with several ml of ultrapure water [54]. The filters were stored at −20°C for 2–6 weeks until spectrophotometric analysis. The amount of Alcian Blue adsorption per sample was determined colorimetrically. Each filter was incubated for 3 h with 6 mL of 80% H_2SO_4 in order to dissolve the particles. The solution was measured at 787 nm with an UV-Vis spectrophotometer (Shimadzu UV-1700 PharmaSpec). The total concentration of TEP is given in µg xanthan gum equivalent (Xeq) L^{-1}, as xanthan gum was used for calibration.

Data analysis and statistics

To determine potential pCO_2/pH effects on TEP concentrations and bacterial abundances, the daily deviation (AD_i) of each mesocosm was calculated by subtracting observations (X_i) from the mean of all mesocosms ($\bar{X}$) on the specific sampling day ($AD_i = X_i - \bar{X}$). These daily deviations were then tested for normality (Shapiro-Wilk) and averaged over time according to $\frac{1}{N}\sum_{i=1}^{N}(AD_i)$ with N being the number of sampling days, in order to get the mean deviations (MD) of each mesocosm regarding a particular parameter [17]. Calculated means and mean deviations were tested against average pH of the different mesocosms by linear regression. Mesocosm M2 was excluded from analysis. Statistical analysis was performed using the software packages Excel and SigmaPlot 12.0 (Systat). Significance was accepted for $p \leq 0.05$.

Results

Plankton growth under pCO_2 perturbation in mesocosms

A first phytoplankton bloom developed in all mesocosms at about day 3 with chlorophyll *a* values increasing up to 3.5 ± 0.3 µg L^{-1} (Fig. 2). The addition of inorganic nutrients (5 µmol L^{-1} nitrate and 0.16 µmol L^{-1} phosphate) to the mesocosms on day 14 initiated the development of a second algal bloom, which reached a maximum of 3.9 ± 0.6 µg Chl *a* L^{-1} between days 19 and 20, declining to 1.4 ± 0.5 µg Chl *a* L^{-1} until day 34 (Fig. 2). In terms of phytoplankton biomass, both blooms

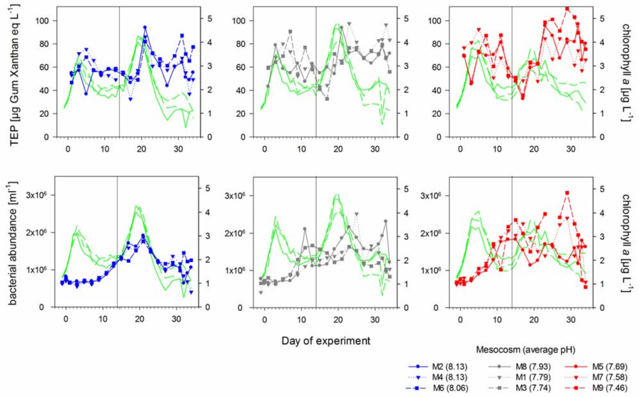

Figure 2. Transparent exopolymer particles and bacterial abundances. Temporal development of transparent exopolymer particles (TEP) concentrations and free-living bacterial abundances in the high (blue), intermediate (grey) and low (red) pH treatments during the course of the experiment. Green lines indicate chlorophyll *a* concentrations in the corresponding mesocosms. Numbers in brackets give the mean pH value of each treatment over time. Vertical black line indicates the day of nutrient addition.
doi:10.1371/journal.pone.0099228.g002

were dominated by chlorophytes and picoeukaryotes (Bermúdez et al., in prep).

The average pH during the phase before nutrient addition ranged between pH 8.13±0.01 in the control mesocosms and pH 7.44±0.30 in the highest pCO_2 mesocosm. After nutrient addition, pH ranged between pH 8.14±0.01 in the control mesocosms and pH 7.49±0.05 in the highest pCO_2 mesocosm (Figure S1). Temperature varied between 6.8°C at the beginning (end of April) and 10.0°C at the end of the experiment in June. During the sampling period, Chl *a* concentrations in the fjord ranged between 0.5 and 1.9 µg L^{-1} (data not shown).

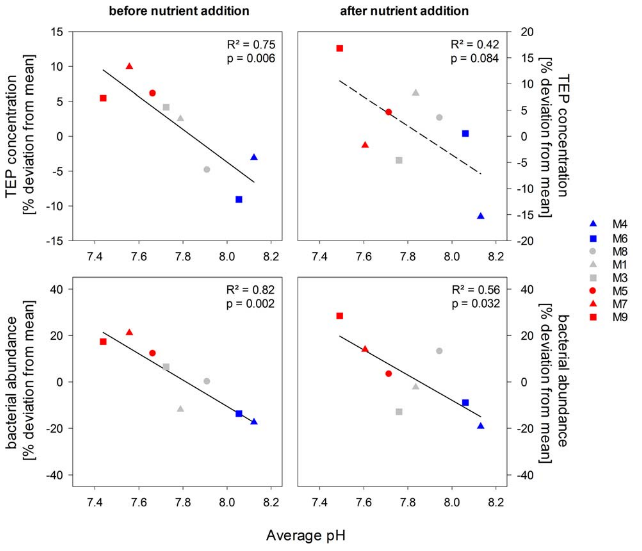

Figure 3. TEP concentrations and bacterial abundances as a function of treatment pH. Symbols indicate the percentage deviation from the mean of all mesocosms over time of TEP and bacterial abundances in the high (blue), intermediate (grey) and low (red) pH mesocosms before and after nutrient addition. Dashed line indicates a non-significant relationship.
doi:10.1371/journal.pone.0099228.g003

Initially, average bacterial cell numbers were $6.5\pm0.9\times10^5$ mL^{-1} (Fig. 2) and increased continuously within the first 25 days to $2.0\pm1.1\times10^6$ cells mL^{-1}. Towards the end of the experiment, bacterial abundance decreased in all treatments to $9.8\pm3.9\times10^5$ mL^{-1} (Fig. 2). Highest bacterial abundance was determined in the high pCO_2/low pH mesocosms with up to 3.1×10^6 cells mL^{-1} on day 29. To compare pCO_2/pH effects at low and higher dissolved nutrient availability, we analysed data of two phases separately: a) before nutrient addition (days 0–13) and b) after nutrient addition (days 14–34). As bacterial abundance may also be regulated by grazing and viral lysis, we refer to changes in bacterial abundances over time as net bacterial growth.

Before nutrient addition, net bacterial growth was faster under elevated CO_2 conditions compared to the control mesocosms ($p=0.002$, Fig. 3) resulting in 12 to 21% significantly higher bacterial abundances in the high pCO_2 mesocosms (Fig. 2) compared to mesocosm average. After nutrient addition, differences in bacterial abundances between treatments were still significant resulting in up to 28% higher bacterial abundance in the highest pCO_2 mesocosm compared to mesocosms average ($p=0.032$, Fig. 3), even though variance between treatments was high. At the same time, variation between treatments increased as biological productivity in the mesocosms proceeded.

Marine gel dynamics

Average TEP concentrations were 59.3±11.2 µg Gum Xanthan equivalent (Xeq) L^{-1} at the beginning of the experiment and increased over time forming two peaks. A first TEP peak was observed at about day 6 with 70.6±13.1 µg Xeq L^{-1}, while the second peak occurred at about day 23 with 81.04±13.6 µg Xeq L^{-1} (Fig. 2). Both TEP peaks occurred two to four days after respective chlorophyll *a* maxima (Fig. 2). In between, TEP concentration decreased to 42.7±9.8 µg Xeq L^{-1}. At the end of the experiment, TEP concentrations in all mesocosms declined to starting values.

A significant treatment effect was detected on TEP concentrations (Fig. 3) before nutrient addition ($p=0.006$). In the low pCO_2 mesocosms, less TEP were measured compared to the over-all mesocosm mean, while in comparison, the high CO_2 mesocosms had up to 5–10% more TEP in the first phase of the experiment. After nutrient addition, differences in TEP concentrations between high and low pCO_2 treatments were not any longer significant as variance between treatments increased ($p=0.084$). TEP concentrations at the end of the experiment were comparable to starting concentrations in all mesocosms indicating that the increased amounts of TEP under high pCO_2/low pH conditions were degraded and/or exported.

Total and cell-specific protein hydrolysis potential

Leucine aminopeptidase (LAP) is a key enzyme during the mineralization of peptides and proteins and widely distributed in the ocean [55]. Therefore, LAP is commonly used as a model enzyme and measured activities give information on the 'protein hydrolysis potential' of the microbial community. LAP activities were low or even not detectable at the beginning of the experiment. During the first two weeks of the experiment, before nutrient addition, inorganic nitrogen became depleted in all mesocosms and total LAP activities increased (Figure S2). After nutrient addition on day 14, LAP activities slightly decreased in all mesocosms but rapidly increased again as soon as the second phytoplankton bloom developed. Maximal activities were measured between day 19 and day 29, when the second phytoplankton bloom declined (Figure S2) yielding an average of 77.2±20.6 nmol L^{-1} h^{-1} equivalent to a substrate turnover time

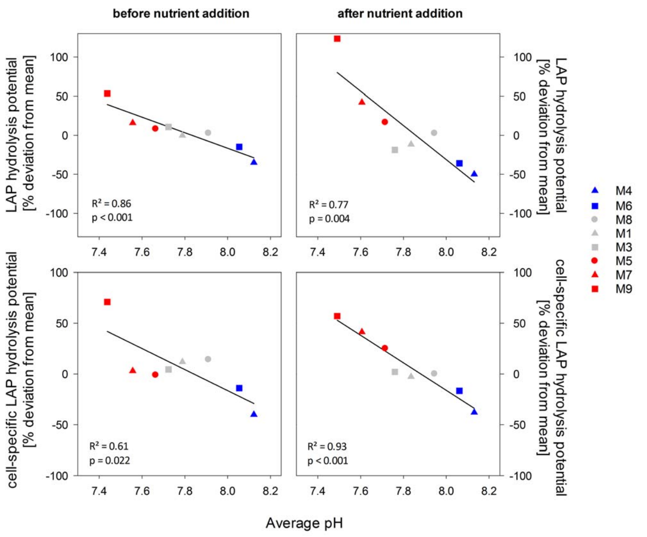

Figure 4. Leucine aminopeptidase (LAP) hydrolysis potential as a function of treatment pH. Symbols indicate the percentage deviation from the mean of all mesocosms over time of total and cell-specific LAP hydrolysis potential in the period before and after nutrient addition in relation to the average pH value. Colors indicate high (blue), intermediate (grey) and low (red) pH treatments.
doi:10.1371/journal.pone.0099228.g004

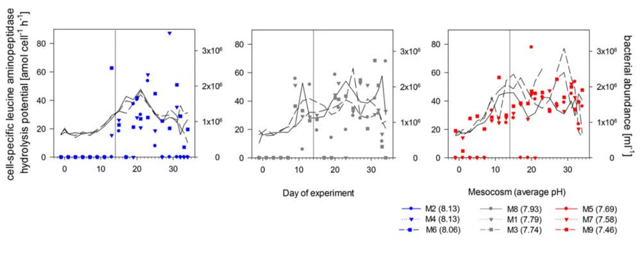

Figure 5. Cell-specific leucine aminopeptidase (LAP) hydrolysis potential. Temporal development of LAP hydrolysis potential in the high (blue), intermediate (grey) and low (red) pH mesocosms during the course of the experiment. Black lines indicate bacterial abundances in the corresponding mesocosms. Numbers in brackets give the mean pH value of each treatment over time. Vertical black line indicates the day of nutrient addition.
doi:10.1371/journal.pone.0099228.g005

of 13.7 h μM^{-1}. Highest LAP activities were measured in the highest pCO_2/lowest pH treatment with 149.2 nmol L^{-1} h^{-1} on day 25. A similar pH dependency was obtained for the mean deviations of total and cell-specific LAP activities. LAP activities increased with decreasing pH also after normalization to bacterial abundance (Fig. 4). This was most pronounced in the highest pCO_2 mesocosm (M9) due to the first 7 days, where only two mesocosms had detectable rates (Fig. 5). Cell-specific activities over the whole experimental period were similar in the low and intermediate pH treatments between pH 8 and 7.7, while the control mesocosms had lowest hydrolysis rates, indicating a direct stimulating effect of lowered pH on protein hydrolysis rates (Fig. 4).

The efficiency of LAP varied over time (Fig. 6) but also largely between treatments while substrate affinity remained relatively constant indicating a non-competitive inhibition of LAP activity by inorganic or organic substances [55]. Before nutrient addition, LAP efficiency was increasing with decreasing pH in the mesocosms resulting in up to three times higher values in the highest pCO_2 mesocosm compared to the controls. After nutrient addition, efficiencies of the enzyme decreased probably due to inhibition by high amounts of inorganic nutrients or freshly produced amino acids as shown in previous studies [55]. In the second post-bloom phase, protein hydrolysis efficiency of the bacterial community was again higher under lower pH conditions (Fig. 6).

Discussion

Impact of changing pH and pCO_2 on TEP formation and leucine aminopeptidase activities

During this mesocosm study, the total amount of TEP was increased under high CO_2 concentrations most likely due to the generally higher biomass production at higher pCO_2 levels. This is in agreement with previous studies showing increased extracellular release of organic compounds by autotrophs under excess availability of CO_2 during nutrient limitation [12,19,56]. Those organic molecules, which are thought to be mostly carbon-rich, are partly consumed by heterotrophic bacteria but also act as precursors for the formation of marine gels such as TEP through coagulation and aggregation of individual colloidal polymers to larger particles. TEP may adsorb additional high amounts of organic and inorganic nutrients and provide a surface for bacteria to attach and grow [57,58]. Consequently, TEP are hotspots for microbial degradation [59] and play an important role in formation of aggregates and export of organic and inorganic matter [60–62].

Our results show that the hydrolysis rates of LAP are accelerated by decreasing seawater pH/increasing pCO_2 confirming previous studies that also reported a stimulation of hydrolytic enzymes such as glucosidases and LAP at high pCO_2 levels [6,37,38,63]. LAP is a widely spread enzyme in marine waters and involved in the decay of proteins providing organic nitrogen to microbes [53]. For bacteria, direct uptake of substrate from seawater is restricted to simple molecules such as mono- and disaccharides and amino acids (LMW-DOM), which are present in very low concentrations in seawater. In contrast, the high-molecular weight (HMW-)DOM fraction needs to be hydrolysed

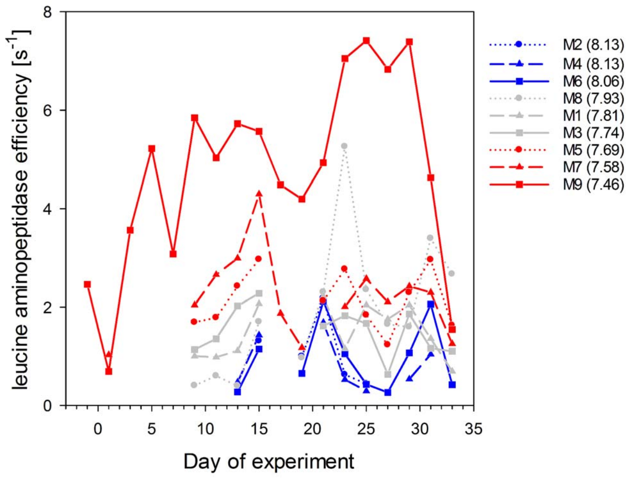

Figure 6. Leucine aminopeptidase efficiency. Temporal development of LAP efficiency (V_{max}:K_m ratio) in the high (red), intermediate (grey) and low (blue) pH mesocosms. Numbers in brackets give the mean pH value of each treatment over time.
doi:10.1371/journal.pone.0099228.g006

prior to microbial uptake. As HMW-DOM contains carbon- and energy-rich substrates, it is turned over more rapidly compared to LMW-DOM [29,30]. The amount and activity of extracellular enzymes determine which molecule can be utilized and how fast organic matter is cycled by heterotrophic microbes. The enzymatic hydrolysis of these substrate molecules is a central step in heterotrophic remineralization [6,34]. As the enzymatic break-down of organic matter seems to become more rapid due to ocean acidification, this might support bacterial nutrition and growth.

Under future ocean conditions, direct and indirect effects of pCO_2 and pH may act in concert and/or even amplify enzymatic hydrolysis of organic matter. Higher cell-specific hydrolysis rates and enzyme efficiencies at lowered pH in our and a previous mesocosm study [64] indicate that seawater acidification might lead to conformational changes in enzyme structure (biochemical effect) accelerating substrate catalysis. In general, attached extracellular enzymes have longer hydrolytic lifetimes than dissolved enzymes [65]. In our study, higher TEP concentrations in the low pH/high CO_2 treatments occurred during and directly after both phytoplankton blooms. More TEP provides more surface area for dissolved enzymes to attach, further increasing the hydrolysis potential of the microbial community (physical effect).

Increased amounts of TEP and faster protein hydrolysis rates support higher bacterial abundance

This study was the second full-scale experiment using this novel sea-going mesocosm system, following an Arctic study in 2010 [41,42]. Our results are the first to show a strong stimulation of the net bacterial growth due to ocean acidification resulting in higher bacterial abundances during and directly after phytoplankton blooms. Potential reasons for the enhanced bacterial success are increased accumulation of gel particles as substrate to attach to and faster nutrient availability due to higher cell-specific protein hydrolysis rates at higher pCO_2/lower seawater pH.

Previous studies implied minor effects of ocean acidification on bacterial abundance [37,66,67]. However, Grossart et al. [37] also reported increased bacterial abundances of particle-attached bacteria at high pCO_2 during the decline of the bloom when the release of algal-derived organic matter was high. We did not determine the abundance of particle-attached bacteria here but as we find higher TEP concentrations, elevated cell numbers in the particulate fraction at high pCO_2 seem likely. Brussaard et al. (2013, [68]) found a stronger regulation of bacterial abundances due to viral lysis at higher pCO_2 during the Arctic mesocosm experiment, although high-CO_2 mesocosms showed a higher protein hydrolysis potential at the start of the experiment. Viral lysis and grazing presumably regulated bacterial abundances to some extent during our study, however, beneficial effects of elevated pCO_2 were more evident.

Bacterial dynamics in the surface ocean are generally tightly coupled to phytoplankton growth and production of labile organic matter. During the first bloom phase autotrophic biomass, based on chlorophyll concentrations, was higher at high pCO_2/low pH levels. This trend reversed during the second bloom (Schulz et al., in prep). At high CO_2 conditions, more carbon- and energy-rich substrates, such as algal exudates due to higher primary production [17] and aggregated polysaccharides in form of TEP, were available for heterotrophic bacteria stimulating microbial growth during the first phase of the experiment.

Inorganic nutrient concentrations limit autotrophic and heterotrophic growth [69], thus microbes are forced to utilize alternative nutrient sources such as atmospheric nitrogen, dissolved organic nitrogen and dissolved organic phosphorus. Before nutrient addition, bacterial abundances at high pH/low pCO_2 increased very slowly presumably due to low availability of inorganic nutrients and labile organic matter in these treatments. In the course of this mesocosm study, enhanced enzymatic hydrolysis of proteins under decreased pH facilitated the microbial acquisition of nutrients from organic matter. We suggest that not only leucine aminopeptidase catalysis is stimulated but also other enzymes involved in carbon- and nutrient cycling may exhibit the same behaviour as shown in previous studies [38,39]. The contribution of bacterial biomass to total biomass in both post-bloom periods was elevated at higher levels of acidification (Figure S3). This stronger stimulation of net bacterial growth compared to autotrophic growth in both phases points towards a direct stimulating effect of pH and pCO_2 on the bacterial community in addition to the effect of increased autotrophic production during the first bloom phase.

It has been stated as null hypothesis that ocean acidification has little effect on microbial driven processes in the oceans because microbial communities always experienced variable pH conditions [70]. In our study, however, we found evidence that marine bacteria thrive under more acidic conditions. The combination of pCO_2 and pH effects, namely (1) increased availability of gel particles as food source and substrate to grow upon and (2) enhanced enzymatic hydrolysis of organic matter, can explain the observed higher bacterial abundances.

Biogeochemical implications

Marine bacteria are involved in all major element cycles and therefore changes in bacterial activities due to climate change may potentially alter marine element fluxes. Increased net bacterial growth based on higher substrate supply may also enhance competition with phytoplankton for inorganic nutrients [27]. In our study, after nutrient addition, phytoplankton biomass was lower under high CO_2/low pH conditions, while bacterial biomass was higher compared to control treatments. As bacteria are very efficient in nutrient acquisition, competition for inorganic nutrient might be one possible reason. In the future ocean, increased bacterial growth in the euphotic zone could thus affect inorganic nutrient concentrations and primary production [6,27].

Whether the surface ocean is a net sink or source of/for atmospheric CO_2 depends amongst other things on the balance of organic matter production and remineralization [10,11]. Microbial respiration provides one of the major natural sources for atmospheric CO_2 [8]. Small changes in microbial growth and degradation rates due to ocean acidification may thus considerably influence atmospheric CO_2 concentrations. Enhanced CO_2 release from marine bacteria in the surface ocean could diminish the ocean's capacity to act as a sink for anthropogenic CO_2 emissions. At the same time, enhanced bacterial remineralization of nutrients would stimulate CO_2 consumption and increase autotrophic production by marine phytoplankton [15,17,23] therefore sequestering more carbon in the ocean. Additionally, higher microbial activity may increase the transformation of labile DOC into refractory DOC that is supposed to be a long-term storage for carbon in the deep sea [33].

Ocean acidification is only one aspect of climate change. Anthropogenic impacts are also expected in terms of temperature, stratification, mixed-layer depth and nutrient availability in the ocean [71]. Similar to acidification, ocean warming was found to increase enzyme activities, bacterial production and respiration rates [6,13,14,72] as well as to enhance polysaccharide release and TEP formation rates [16,73]. We therefore hypothesize that ocean acidification and warming act in concert to intensify heterotrophic

microbial processes and to reinforce the already central role of bacteria in the cycling of organic matter in the ocean.

Supporting Information

Figure S1 pH values in the nine mesocosms over time. Mesocosms were adjusted until day 5 to target pCO_2 levels by stepwise additions of CO_2 saturated seawater. Nutrients were added to all mesocosms on day 14.
(TIF)

Figure S2 Leucine aminopeptidase (LAP) hydrolysis potential. Temporal development of total LAP hydrolysis potential in the high (blue), intermediate (grey) and low (red) pH mesocosms during the course of the experiment. Black lines indicate bacterial abundances in the corresponding mesocosms. Numbers in brackets give the mean pH value of each treatment over time. Vertical black line indicates the day of nutrient addition.
(TIF)

Figure S3 Percentage of bacterial biomass in particulate organic carbon over time in high (blue), intermediate (grey) and low (red) pH mesocosms. Green lines indicate chlorophyll *a* concentrations in the corresponding mesocosms. Vertical black line indicates the day of nutrient addition.
(TIF)

Acknowledgments

We thank all participants of the Bergen Mesocosm Experiment 2011, in particular A. Ludwig, J. Czerny, M. Meyerhöfer, the KOSMOS team, and the staff at the Marine Biological Station, University of Bergen for providing mesocosm logistics, technical assistance and support during sampling. S. Koch-Klavsen is gratefully acknowledged for measuring chlorophyll concentrations. We are grateful for the technical support of J. Roa, A. Paul and C. Mages. We thank the captains and crews of R/V Håkon Mosby, R/V Alkor and R/V Heincke for support during transport, deployment and recovery of the mesocosm facility. We appreciated the constructive comments of two anonymous referees and the editor.

Author Contributions

Conceived and designed the experiments: UR AE. Performed the experiments: SE LG KGS. Analyzed the data: SE LG UR KGS AE. Contributed reagents/materials/analysis tools: UR KGS AE. Wrote the paper: SE LG UR KGS AE.

References

1. Cho BC, Azam F (1988) Major Role of Bacteria in Biogeochemical Fluxes in the Oceans Interior. Nature 332 (6163): 441–443.
2. Azam F, Smith DC, Steward GF, Hagström A (1993) Bacteria-organic matter coupling and its significance for oceanic carbon cycling. Microbial Ecology 28: 167–179.
3. Smith DC, Simon M, Alldredge AL, Azam F (1992) Intense hydrolytic enzyme activity on marine aggregates and implications for rapid particles dissolution. Nature 359: 139–142.
4. Sabine CL (2004) The Oceanic Sink for Anthropogenic CO_2. Science 305 (5682): 367–371.
5. Wolf-Gladrow DA, Riebesell U, Burkhardt S, Bijma J (1999) Direct effects of CO_2 concentration on growth and isotopic composition of marine plankton. Tellus B 51B (2): 461–476.
6. Cunha A, Almeida A, Coelho FJRC, Gomes NCM, Oliveira V, et al. (2010) Bacterial Extracellular Enzymatic Activity in Globally Changing Aquatic Ecosystems. In: Méndez-Vilas A, editor. Current research, technology and education topics in applied microbiology and microbial biotechnology. Badajoz, Spain: Formatex Research Center. pp. 124–135.
7. del Giorgio PA, Duarte CM (2002) Respiration in the open ocean. Nature 402: 379–384.
8. Martin JH, Knauer GA, Karl DM, Broenkow WW (1987) VERTEX: carbon cycling in the northeast Pacific. Deep Sea Research 34 (2): 267–285.
9. Kirchman DL, Morán XAG, Ducklow H (2009) Microbial growth in the polar oceans — role of temperature and potential impact of climate change. Nature Reviews Microbiology 7: 451–458.
10. Suess E (1980) Particulate organic carbon flux in the oceans - surface productivity and oxygen utilization. Nature 288 (5788): 260–263.
11. Passow U, Carlson CA (2012) The biological pump in a high CO_2 world. Marine Ecology Progress Series 470: 249–271.
12. Engel A (2002) Direct relationship between CO_2 uptake and transparent exopolymer particles production in natural phytoplankton. Journal of Plankton Research 24 (1): 49–53.
13. Piontek J, Händel N, Langer G, Wohlers J, Riebesell U, et al. (2009) Effects of rising temperature on the formation and microbial degradation of marine diatom aggregates. Aquatic Microbial Ecology 54: 305–318.
14. Wohlers J, Engel A, Zollner E, Breithaupt P, Jürgens K, et al. (2009) Changes in biogenic carbon flow in response to sea surface warming. Proceedings of the National Academy of Sciences of the United States of America 106 (17): 7067–7072.
15. Riebesell U, Kortzinger A, Oschlies A (2009) Sensitivities of marine carbon fluxes to ocean change//Tipping Elements in Earth Systems Special Feature: Sensitivities of marine carbon fluxes to ocean change. Proceedings of the National Academy of Sciences of the United States of America 106 (49): 20602–20609.
16. Borchard C, Engel A (2012) Organic matter exudation by *Emiliania huxleyi* under simulated future ocean conditions. Biogeosciences 9 (8): 3405–3423.
17. Engel A, Borchard C, Piontek J, Schulz KG, Riebesell U, et al. (2013) CO_2 increases ^{14}C primary production in an Arctic plankton community. Biogeosciences 10 (3): 1291–1308.
18. Passow U (2002) Transparent exopolymer particles (TEP) in aquatic environments. Progress In Oceanography 55 (3–4): 287–333.
19. Engel A, Delille B, Jacquet S, Riebesell U, Rochelle-Newall E, et al. (2004) Transparent exopolymer particles and dissolved organic carbon production by *Emiliania huxleyi* exposed to different CO_2 concentrations: a mesocosm experiment. Aquatic Microbial Ecology 34: 93–104.
20. Nagata T (2008) Organic matter-bacteria interactions in seawater. In: Kirchman DL, Kirchman DL, editors. Microbial ecology of the oceans. 2nd edition. New Jersey: Wiley-Blackwell. pp. 207–241.
21. Beardall J, Raven JA (2004) The potential effects of global climate change on microalgal photosynthesis, growth and ecology. Phycologia 43 (1): 26–40.
22. Schippers P, Lurling M, Scheffer M (2004) Increase of atmospheric CO_2 promotes phytoplankton productivity. Ecology letters 7 (6): 446–451.
23. Riebesell U, Tortell PD (2011) Effects of Ocean Acidification on Pelagic Organisms and Ecosystems. In: Gattuso J, Hansson L, editors. Ocean acidification. Oxford [England], New York: Oxford University Press.
24. del Giorgio PA, Cole JJ (1998) Bacterial growth efficiency in natural aquatic systems. Annual Review of Ecology and Systematics 29: 503–541.
25. Azam F (1998) Microbial control of oceanic carbon flux: The plot thickens. Science 280 (5364): 694–696.
26. Lovdal T, Eichner C, Grossart HP, Carbonnel V, Chou L, et al. (2008) Competition for inorganic and organic forms of nitrogen and phosphorous between phytoplankton and bacteria during an *Emiliania huxleyi* spring bloom. Biogeosciences 5 (2): 371–383.
27. Thingstad TF, Bellerby RG, Bratbak G, Borsheim KY, Egge JK, et al. (2008) Counterintuitive carbon-to-nutrient coupling in an Arctic pelagic ecosystem. Nature 455 (7211): 387–390.
28. Chrost RJ, Münster U, Rai H, Albrecht D, Witzel PK, et al. (1989) Photosynthetic production and exoenzymatic degradation of organic matter in the euphotic zone of a eutrophic lake. Journal of Plankton Research 11 (2): 223–242.
29. Amon RMW, Benner R (1994) Rapid cycling of high-molecular-weight dissolved organic matter in the ocean. Letters to Nature 369: 549–552.
30. Amon RMW, Benner R (1996) Bacterial utilization of different size classes of dissolved organic matter. Limnology and Oceanography 41 (1): 41–51.
31. Kattner G, Simon M, Koch BP (2011) Molecular Characterization of Dissolved Organic Matter and Constraints for Prokaryotic Utilization. In: Jiao N, Azam F, Sanders S, editors. Microbial Carbon Pump in the Ocean: The American Association for the Advancement of Science. pp. 60–61.
32. Ogawa H, Amagai Y, Koike I, Kaiser K, Benner R (2001) Production of refractory dissolved organic matter by bacteria. Science 292 (5518): 917–920.
33. Jiao N, Herndl GJ, Hansell DA, Benner R, Kattner G, et al. (2010) Microbial production of recalcitrant dissolved organic matter: long-term carbon storage in the global ocean. Nature Reviews Microbiology 8 (8): 593–599.
34. Chrost RJ (1992) Significance of Bacterial Ectoenzymes in Aquatic Environments. Hydrobiologia 243: 61–70.
35. Tipton KF, McDonald AG, Dixon HBF (2009) Effect of pH on Enzymes. In: Purich DL, editor. Contemporary enzyme kinetics and mechanism. Amsterdam, Boston: Elsevier/Academic Press.
36. Yamada N, Suzumura M (2010) Effects of Seawater Acidification on Hydrolytic Enzyme. Journal of Oceanography 66 (2): 233–241.

37. Grossart H, Allgaier M, Passow U, Riebesell U (2006) Testing the effect of CO_2 concentration on the dynamics of marine heterotrophic bacterioplankton. Limnology and Oceanography 51 (1): 1–11.
38. Piontek J, Lunau M, Händel N, Borchard C, Wurst M, et al. (2010) Acidification increases microbial polysaccharide degradation in the ocean. Biogeosciences 7 (5): 1615–1624.
39. Tanaka T, Thingstad TF, Lovdal T, Grossart H-, Larsen A, et al. (2008) Availability of phosphate for phytoplankton and bacteria and of glucose for bacteria at different pCO_2 levels in a mesocosm study. Biogeosciences 5: 669–678.
40. Maas EW, Law CS, Hall JA, Pickmere S, Currie KI, et al. (2013) Effect of ocean acidification on bacterial abundance, activity and diversity in the Ross Sea, Antarctica. Aquatic Microbial Ecology 70 (1): 1–15.
41. Riebesell U, Czerny J, Bröckel K von, Boxhammer T, Büdenbender J, et al. (2013) Technical Note: A mobile sea-going mesocosm system – new opportunities for ocean change research. Biogeosciences 10: 1835–1847.
42. Schulz KG, Bellerby RGJ, Brussaard CPD, Büdenbender J, Czerny J, et al. (2013) Temporal biomass dynamics of an Arctic plankton bloom in response to increasing levels of atmospheric carbon dioxide. Biogeosciences 10 (1): 161–180.
43. Dickson AG (2010) The carbon dioxid system in seawater: equilibrium chemistry and measurments. In: Riebesell U, Fabry VJ, Hansson L, Gattuso JP, editors. Guide to best practise in ocean acidification research and data reporting. Luxembourg. pp. 17–40.
44. Welschmeyer NA (1994) Fluorometric Analysis of Chlorophyll-A in the Presence of Chlorophyll-B and Pheopigments. Limnology and Oceanography 39 (8): 1985–1992.
45. Gasol JM, del Giorgio PA (2000) Using flow cytometry for counting natural planktonic bacteria and understanding the structure of planktonic bacterial communities. Scientia Marina 64 (2): 197–224.
46. Turley CM, Hughes DJ (1994) Effect of storage temperature on enumeration of epifluorescence-detectable bacterial cells in preserved seawater samples. Journal of the Marine Biological Association of the United Kingdom 74: 259–262.
47. Parvathi A, Radhakrishnan S, Sajila MP, Jacob B (2011) Study of changes in bacterial and viral abundance in formaldehyde-fixed water samples by epifluorescence microscopy. Environmental Monitoring and Assessment 177 (1–4): 227–231.
48. Troussellier M, Courties C, Zettelmaier S (1995) Flow Cytometric Analysis of Coastal Lagoon Bacterioplankton and Picophytoplankton: Fixation and Storage Effects. Estuarine, Coastal and Shelf Science 40 (6): 621–633.
49. Lunau M, Lemke A, Walther K, Martens-Habbena W, Simon M (2005) An improved method for counting bacteria from sediments and turbid environments by epifluorescence microscopy. Environmental Microbiology 7 (7): 961–968.
50. Fagerbakke KM, Heldal M, Norland S (1996) Content of carbon, nitrogen, oxygen, sulfur and phosphorus in native aquatic and cultured bacteria. Aquatic Microbial Ecology 10: 15–27.
51. Caruso G, Zaccone R (2000) Estimates of leucine aminopeptidase activity in different marine and brackish environments. Journal of Applied Microbiology 89: 951–959.
52. Hoppe H (1983) Significance of exoenzymatic activities in the ecology of brackish water: measurements by means of methylumbelliferyl-substrates. Marine Ecology Progress Series 11: 299–308.
53. Chróst RJ, Siuda W (2002) Ecology of Microbial Enzymes in Lake Ecosystems. In: Burns RG, Dick RP, editors. Enzymes in the environment. Activity, ecology, and applications. New York: Marcel Dekker. pp. 35–72.
54. Passow U, Alldredge AL (1995) A dye-binding assay for the spectrophotometric measurement of transparent exopolymer particles (TEP). Limnology and Oceanography 40 (7): 1326–1335.
55. Chrost RJ (1991) Environmental Control of the Synthesis and Activity of Aquatic Microbial Ectoenzymes. In: Chrost RJ, editor. Microbial Enzymes in Aquatic Environments. New York: Brock/Springer Series in Contemporary Bioscience.
56. Obernosterer I, Herndl GJ (1995) Phytoplankton Extracellular Release and Bacterial-Growth - Dependence on the Inorganic N-P Ratio. Marine Ecology Progress Series 116 (1–3): 247–257.
57. Long RA, Azam F (1996) Abundant protein-containing particles in the sea. Aquatic Microbial Ecology 10: 213–221.
58. Azam F, Malfatti F (2007) Microbial structuring of marine ecosystems. Nature Reviews Microbiology 5 (10): 782–791.
59. Verdugo P, Alldredge AL, Azam F, Kirchman DL, Passow U, et al. (2004) The oceanic gel phase: a bridge in the DOM-POM continuum. Marine Chemistry 92 (1–4): 67–85.
60. Passow U, Shipe R, Murray A, Pak D, Brzezinski M, et al. (2001) The origin of transparent exopolymer particles (TEP) and their role in the sedimentation of particulate matter. Continental Shelf Research 21 (4): 327–346.
61. Engel A, Thoms S, Riebesell U, Rochelle-Newall E, Zondervan I (2004) Polysaccharide aggregation as a potential sink of marine dissolved organic carbon. Nature 428: 929–932.
62. Engel A, Piontek J, Grossart H, Riebesell U, Schulz KG, et al. (2014) Impact of CO_2 enrichment on organic matter dynamics during nutrient induced coastal phytoplankton blooms. Journal of Plankton Research 0 (0): 1–17.
63. Endres S, Unger J, Wannicke N, Nausch M, Voss M, et al. (2013) Response of *Nodularia spumigena* to pCO_2 – Part 2: Exudation and extracellular enzyme activities. Biogeosciences 10 (1): 567–582.
64. Piontek J, Borchard C, Sperling M, Schulz KG, Riebesell U, et al. (2013) Response of bacterioplankton activity in an Arctic fjord system to elevated pCO_2: results from a mesocosm perturbation study. Biogeosciences 10 (1): 297–314.
65. Ziervogel K, Karlsson E, Arnosti C (2007) Surface associations of enzymes and of organic matter: Consequences for hydrolytic activity and organic matter remineralization in marine systems. Marine Chemistry 104 (3–4): 241–252.
66. Rochelle-Newall E, Delille B, Frankignoulle M, Gattuso JP, Jacquet S, et al. (2004) Chromophoric dissolved organic matter in experimental mesocosms maintained under different pCO_2 levels. Marine Ecology Progress Series 272: 25–31.
67. Allgaier M, Riebesell U, Vogt M, Thyrhaug R, Grossart HP (2008) Coupling of heterotrophic bacteria to phytoplankton bloom development at different pCO_2 levels: a mesocosm study. Biogeosciences 5 (4): 1007–1022.
68. Brussaard CPD, Noordeloos AAM, Witte H, Collenteur MCJ, Schulz K, et al. (2013) Arctic microbial community dynamics influenced by elevated CO_2 levels. Biogeosciences 10 (2): 719–731.
69. Kirchman DL (1994) The uptake of inorganic nutrients by heterotrophic bacteria. Microbial Ecology 28: 255–271.
70. Joint I, Doney SC, Karl DM (2010) Will ocean acidification affect marine microbes. The ISME Journal 5 (1): 1–7.
71. Boyd PW (2011) Beyond ocean acidification. Nature Geoscience 4 (5): 273–274.
72. Wohlers-Zollner J, Breithaupt P, Walther K, Jurgens K, Riebesell U (2011) Temperature and nutrient stoichiometry interactively modulate organic matter cycling in a pelagic algal-bacterial community. Limnology and Oceanography 56 (2): 599–610.
73. Engel A, Handel N, Wohlers J, Lunau M, Grossart H, et al. (2011) Effects of sea surface warming on the production and composition of dissolved organic matter during phytoplankton blooms: results from a mesocosm study. Journal of Plankton Research 33 (3): 357–372.
